# Supplementary material for: Photo-excited charge carriers suppress sub-terahertz phonon mode in silicon at room temperature
Source: Nat Commun. 2016 Oct 12;7:13174. doi: 10.1038/ncomms13174 (PMC5064017; doi:10.1038/ncomms13174)
Supplement: Supplementary Information — Supplementary Figure 1 and Supplementary Table 1. [file ncomms13174-s1.pdf]

Supplementary Information

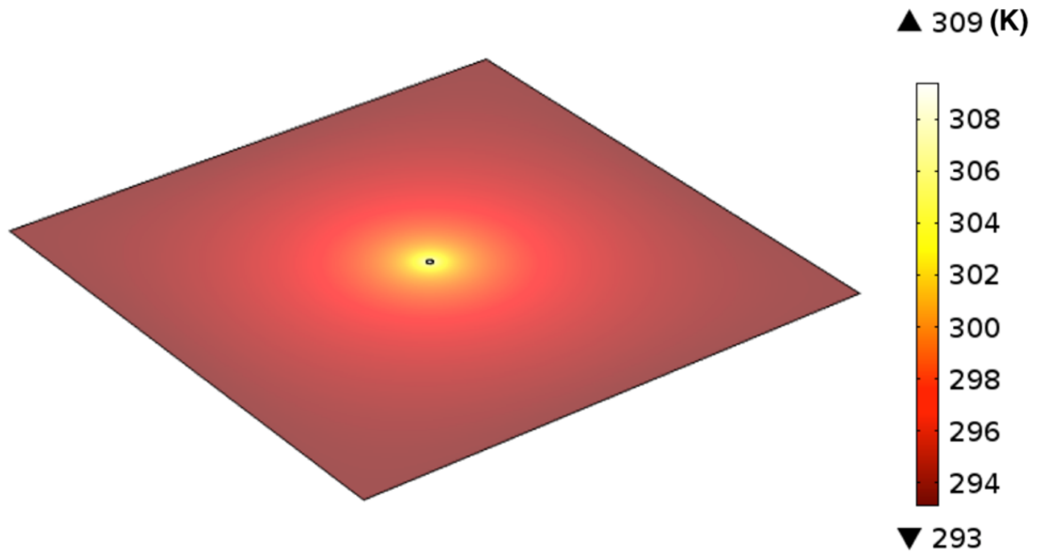

Supplementary Figure 1. COMSOL simulation of the steady-state temperature rise of the silicon membrane under illumination of 15 mW of 780-nm excitation beam with 60-μm beam diameter. The size of the membrane is 5 mm by 5 mm, and the thickness is 1.7 μm. The membrane is supported by a 500-μm-thick silicon frame, which is set at room temperature (293 K) in the simulation. The upper and lower surfaces of the membrane are set as adiabatic in the simulation. Since there is natural convection and radiation from the surfaces in the real experiment, the actual temperature rise should be lower than the simulation.

400 nm Pump Power (mW)	6	9	12	15
Intrinsic Lifetime (ps)	238.3±57.9	224.9±26.1	234.3±23.5	248.2±3.5

Supplementary Table 1. The measured phonon lifetime without the 780-nm excitation beam with different power of the 390-nm pump pulses, at the same location on the membrane. There is no systematic reduction of the intrinsic phonon lifetime with increasing pump power observed. The large uncertainty at lower pump power is due to the reduced signal amplitude and thus the signal-noise ratio.
